# How context affects people’s willingness to register for the deceased organ donation programme

**DOI:** 10.1186/s12889-021-10753-5

**Published:** 2021-04-15

**Authors:** Lamia AlHajri, Amna AlHebsi, Maryam AlSuwaidi

**Affiliations:** 1 Department of Health Sciences, Higher Colleges of Technology, Abu Dhabi, United Arab Emirates; 2 Department of Health Research, Lancaster University, Lancashire, UK

**Keywords:** Deceased organ transplantation, Qualitative, Experience, UAE

## Abstract

**Background:**

The deceased organ donation programme is new in the United Arab Emirates (UAE), and to improve acceptability, a broad understanding of public perspectives is thought to be helpful. Therefore, this study aims to explore the extent to which context plays a role in the willingness to register for the deceased organ donation programme in Dubai, UAE.

**Methods:**

This study used a qualitative methodology and was gauged by the tenets of a social ecological model and lay knowledge. Audio-recorded semi-structured interviews were conducted with 17 participants. The data were subsequently analysed thematically.

**Results:**

Four themes emerged from the dataset: fear and body integrity, family, relational ties and the identification of the recipient, religious conviction, and knowledge and personal experiences. The participants feared the whole process, were not aware of the religious outlook, and their knowledge regarding the programme was scarce. In addition, family-related factors, such as parental authority and hierarchy in the family, were also major influencers.

**Conclusion:**

Using the social ecological model and lay knowledge helped to unravel the contextual factors that affected the willingness of participants to register for the deceased organ donation programme in Dubai, UAE, thereby enabling the development of a holistic understanding of deceased organ donation. The responses mainly stemmed from participants’ social contexts; hence, awareness campaigns should be tailored to inform people about the technical aspects and address their contextual concerns.

**Supplementary Information:**

The online version contains supplementary material available at 10.1186/s12889-021-10753-5.

## Background

Transplantation is the greatest and only life-saving strategy for patients with end-stage organ failure [1, 2]. Studies have shown that transplantation improves survival rates and quality of life [3]. Alongside the advancements in surgical techniques and the availability of effective immunosuppressive agents, deceased organ donation programmes are an important addition in making organ transplantation more accessible to patients with organ failure [1]. Studies found that there are more than 6000 patients per year waiting for a transplant, with a 10 to 30% chance of dying while waiting for organ transplantation [1]. Spain has been one of the leading countries in implementing the deceased organ donation programme for more than 25 years, with a total of 4818 organ transplantations carried out during 2016 [4]. This could be primarily due to the opt-out system, in which every deceased person is automatically recognised as a potential organ donor unless they sign an opt-out [4].

**Fig. 1:**
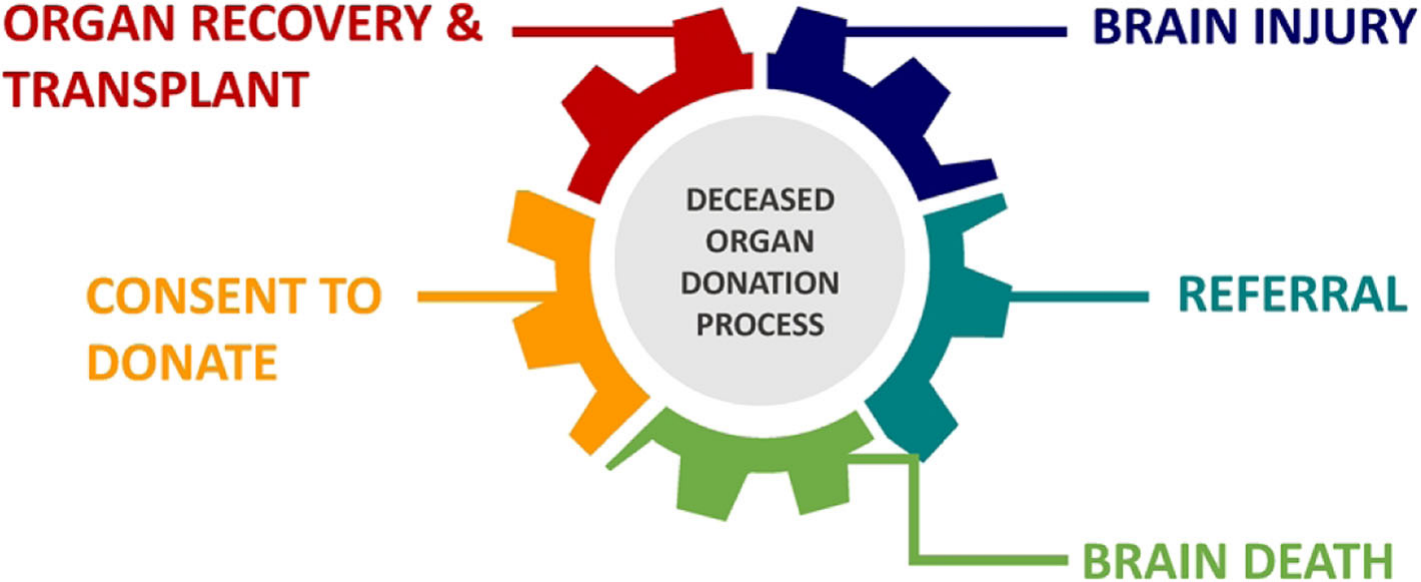
The process of deceased organ donation [1, 10]

Given the pressing need, the shortage of organs, and the importance of organ donation in saving the lives of patients, the late Sheikh Zayed Bin Sultan started paving the way for organ transplantation in the United Arab Emirates (UAE) in 1993 [5]. However, it was only in 2010 that the first kidney transplant for an Emirati patient took place in Abu Dhabi, UAE [6]. And in May 2013, the first successful transplantation from a deceased person was performed in the UAE [7]. In 2016, the Federal Decree-Law No. 5 of 2016 on Regulation of Human Organs and Tissue Transplantation allowing and regulating organ transplantation between live and deceased donors was implemented [8]. Since then, 6 deceased patients in the UAE have donated their organs to save the lives of 22 patients (12 kidneys, 3 livers, 4 lungs, 2 hearts, and a pancreas) [9]. The deceased organ donation process involves six stages: brain injury, referral, brain death, consent, and organ recovery and transplant, as shown in Fig. 1 [1, 10]. Stage 4 is concerned with obtaining consent to donate from either the donor before death or from the grieving families [1, 10]. However, a number of studies brought to light that registering for the deceased organ donation programme is far from simple [11–15].

There are plenty of contextual and situation-specific factors that interplay in a complex character, affecting people’s willingness to register for such a programme. For example, family ties, parental approval, religious and cultural conflicts, and much more were major players in making such a decision, based on studies conducted in Saudi Arabia, Israel, Iran, and Australia [11–15]. Patients, and people in general, are complex; therefore, in the sensitive topic of ‘deceased organ donation’, the focus should go beyond the biological aspect and include the social determinants, environment, and social relations, elements that are intricately entangled [16]. However, to be able to understand these subjective contextual factors, lay knowledge has to be unravelled. Lay knowledge is patient-specific and is developed through the lived patients’ experiences with the disease within their social contexts [17, 18]. It is driven by subjective personal concerns, life situations, and surrounding contexts [19, 20]. As the deceased organ donation programme is new in the UAE, a broad understanding of lay-public views may help to identify and explain any barriers to participation to improve the organ donation culture in society and render the programme more successful. Although plenty of studies have explored people’s perceptions towards organ donation or deceased organ donation, none were conducted in the UAE or examined to what extent context plays a role in participants’ willingness to register for the deceased organ donation programme in Dubai, UAE, gaps in the literature that this study aims to address.

### Theoretical and conceptual framework

We adopt the social ecological model (SEM), which focuses on exploring, understanding, and addressing the multifaceted and interactive effects of personal, cultural, social and environmental factors (context) on behaviour [21, 22]. The SEM has five hierarchical levels—individual, interpersonal, community, organizational, and policy/enabling environment—and places a great deal on the interdependence and interconnection between these levels and their constituent factors [21, 22]. Therefore, this model encourages going beyond the biological aspect or considering individuals and their consciousness as the only controllers of actions or behaviours towards understanding the wide range of factors that influence a certain behaviour or action [21, 22]. Hence, the SEM allows the identification of a wider range of contextual subjective factors and influences while appreciating their interactive nature and that individuals are embedded within larger systems [22–26]. To develop a better understanding of these contextual and situational factors, people with first-hand experience with their context should be involved. The aim is to obtain lay knowledge, which allows us to bring to practice patients’ experiences in context, which is subjective and diverse and stems from natural settings [20]. Using these theoretical and conceptual frameworks enhances our understanding of how contexts affect people’s willingness to sign up for the deceased organ donation programme.

## Methods

### Philosophical paradigm

A qualitative methodology underpinned by an interpretivist paradigm with both subjective epistemology and relativist ontological stances was adopted [27–30]. An interpretivist approach was thought to be the most suitable, as this study is mainly aimed at unravelling participants’ lay knowledge regarding their contexts and views of the deceased organ donation programme, which were found to be diverse, multiple, subjective, and complex, as seen in the results section [27–29, 31]. This philosophical approach and methodology enable the inductive development of a deep understanding of the phenomenon under investigation from the participants’ perspective, which is important for achieving the aim of this research [30]. In addition, a qualitative methodology provides flexibility and preserves natural settings, increasing the chance of capturing the complexity of the phenomenon [27, 32, 33].

### Recruitment

To collect data that would help answer the research question, purposive, voluntary, and snowball sampling methods were used, as delineated in this section [29, 31]. This is because the type of knowledge and reality we aim to become acquainted with resides within people who are interacting with their context and have heard about the UAE deceased organ donation programme, have experienced it, or witnessed someone experiencing it or a similar programme [29, 31]. Since the deceased organ donation programme is still relatively new in the UAE, the researchers faced some difficulties with recruitment; therefore, snowball sampling was used as well. The researchers (all females) recruited the participants by posting an invitation on various social media platforms (Instagram, Twitter, Facebook, and LinkedIn). Ten subjects were initially selected, and another seven joined via snowball sampling. Although this was a limitation of our recruitment process, the point of saturation was reached at the fifteenth participant, yet another two were interviewed to ensure that the recruitment process was not halted prematurely. The same obstacle was observed in other studies, where the response rate was low during the recruitment phases [34, 35]. Regarding the sample size, there was no specific consensus about the number of participants, as it could vary between 1 and 325 [36, 37]. Although the phenomenon under investigation is highly sophisticated and diverse, having to remain pragmatic is crucial [38, 39]. During selection, maximum variation was maintained to ensure a range of participants, thereby capturing a diversity of perspectives [31]. Hence, individuals of different ages, nationalities, ethnicities, and religions were recruited. All participants were given an information sheet and signed a consent form before taking part in the study, as described in the ethics section below.

Audio-recorded semi-structured interviews about 60 min long were conducted using a topic guide derived from the literature and SEM (Additional file 1). The semi-structured format provides data with great depth and breadth and allows participants to narrate their experiences without being tied to specific answers, which is important for this research [40, 41]. This approach ensured flexibility while maintaining focus on the topic [42]. Audio-recording was used because it enables to capture linguistic as well as non-linguistic findings by allowing researchers to take notes of body language. The location, date, and timing of the interviews were chosen according to the participant’s preferences. However, the chosen locations had to be safe, comfortable, private, and free of distractions. The interviews were conducted in different locations, ranging between coffee shops and the homes of participants. A trial interview using the topic guide was conducted with four participants to ensure that the topic guide would capture the required data and was clear, understandable, and free of jargon. These individuals were excluded from the actual study. Additionally, individuals’ identities were concealed using codes (letters followed by numbers), which guaranteed data anonymity and confidentiality. During the interviews, the participants were first allowed to settle while confirming the demographic information and filling out the consent form, if not done earlier. Next, the completely anonymised recording was started. Researchers ensured that they write a reflection after each interview within no more than an hour. This helped to enrich findings and understanding of the engendered data.

### Ethical considerations

The subjects were fully informed about the purpose, methods, and intended possible uses of the data, what their participation in the study entailed, and what risks, if any, were involved. The participants were provided with an information sheet, including information about the voluntary nature of this study and their right to withdraw from it at any given time. In this participant information sheet, it was also mentioned that the findings would be submitted for publication and that their names would be replaced with codes. The computers holding data were password-protected, and the audio files were password-protected and encrypted. The audio-recorded files were destroyed immediately after the completion of this study. Any paper that might have contained personal identifiers was locked in a cabinet.

### Data analysis

#### Thematic analysis

After transcribing the verbal data, thematic analysis was carried out as described in Table 1 [43] using Nvivo. This type of analysis helps identify, analyse, and report patterns (themes) and interpret the data [43].

**Table 1:** Phases of thematic analysis [43]

	Steps
1.	Becoming familiar with the data
2.	Generating initial codes
3.	Searching for themes
4.	Reviewing themes
5.	Defining and naming themes
6.	Interpretative analysis

#### Validity and reliability

As the researcher is the primary data collection tool, measures were implemented to maintain the authenticity of the findings and avoid imposing the researcher’s perspectives, which might alter the findings [29]. Reflexivity, audit trails, member checks, participant verifications, thick descriptions, and more were used to enhance rigour, as delineated in Table 2 [31]. The recordings were transcribed and interpreted immediately (within 2 days of conducting the interview) to ensure that details about the interviews were adequately documented and to introduce any necessary amendments to the topic guide to retrieve more purposive information [27].

**Table 2:** Rigour assessment and assurance techniques [29]

Rigour assessment parameter	Techniques used to enhance rigour
1) Credibility	• Member checks: researchers’ and participant’s verification of interpretation • Reflexivity and audit trail: the three researchers reflected on how they affected and were affected by the research and how all decisions were made. • Thick descriptions • Examine previous research. • Competency of researchers: one of the researchers was the research supervisor.
2) Transferability	• Thick descriptions • Maximum variations: selecting a sample that encompasses a wide range of cases
3) Consistency or dependability	• Peer examination: the researchers verified for each other. • Reflexivity and audit trail
4) Confirmability	• Reflexivity and audit trail

## Results

### Demographic information

The demographic information is summarised in Table 3. All participants were either currently living or had lived in Dubai. A proportion of 58.8% of the sample were female and 41.2% male. The participants were of nine nationalities and different age groups, educational levels, religious beliefs, and marital statuses. This variability in demographics ensured a wide range of perspectives on deceased organ donation.

**Table 3:** Characteristics of demographic information

Demographic Characteristic		Number
Nationality	UAE	7
	India	2
	Pakistan	1
	Philippine	2
	Jordan	1
	Egypt	1
	Iraq	1
	United Kingdom	1
	Palestine	1
Gender	Male	7
	Female	10
Age (Year)	18–29	6
	30–45	7
	46–60	3
	> 60	1
Marriage	Married	11
	Single	6
Education	Bachelor	15
	Higher Degree	2
Religion belief	Christian	3
	Hinduism	2
	Islam	12

### Themes

**Fig. 2:**
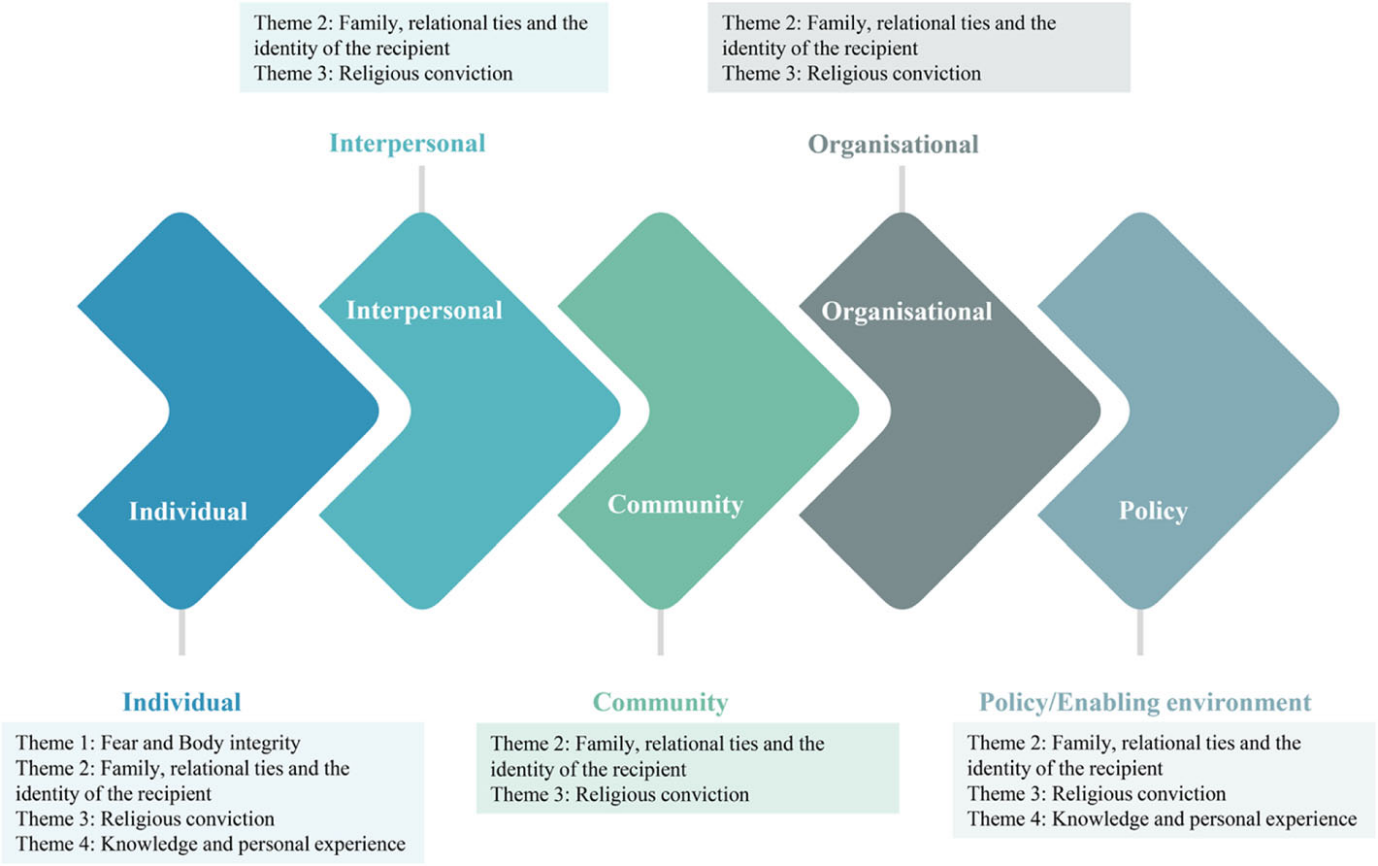
The social ecological model

The data were explored through the SEM lens, resulting in the identification of four themes, as delineated below and in Fig. 2.

1. Fear and body integrity
2. Family, relational ties, and the identity of the recipient
3. Religious convictions
4. Knowledge and personal experience.

#### First theme: fear and body integrity

Fear, which mainly stemmed from the SEM’s individual level, was a major factor in deciding whether a person would sign up for the deceased organ donation programme. All participants talked about their fear of registration. The majority mentioned that it was actually their fear of pain, harm, and of the unknown. Some participants stated that the word ‘deceased’ reminded them of death and that the human body is a sanctifying component for many individuals’; therefore, deceased organ donation was perceived as an action that dehumanises or imperils the dignity, identity, or individuality of the body.

*‘It scares me. Whenever I see this form, I feel as if I am signing on my death’. (Participant 13, female, 37 years old)*

*‘Someone told me that basically, the body feels after death, so you might feel that you are being cut up.. and that.. that scares me. When I die, would I want to feel pain even after I die? NO!’ (Participant 9, male, 26 years old)*

*‘I do not want my body to be cut open and take stuff from me after my death. Skin, heart, bones, everything, everything, they are mine!’ (Participant 6, male, 22 years old)*

The participants also talked about the importance of providing care to patients to prevent death. However, due to their mistrust of the healthcare system, they feared that interventions might be withheld so that patients would become eligible for the deceased organ donation programme. Another source of worry was that their organs would be harvested unethically or before they died to be given to specific high-profile people due to lobbying.

*‘I do not trust them to do their best in saving my life if they knew that I have registered for organ donation. I prefer to die and take everything with me to the grave’. (Participant 2, female, 42 years old)*

On the other hand, some participants had the opposite perception: they thought of the programme as essential for the survival of those in need, that they had nothing to lose from the donation, and some even perceived it as a ‘noble’ act (participant 6, male, 22 years old).

*‘I am not gonna lose anything. I am dead already. Instead so many people might have a better quality of life because of my donation’. (Participant 7, female, 19 years old)*

#### Second theme: family, relational ties, and the identity of the recipient

Family, relational ties, and the recipient’s identity constituted a theme that emerged from all levels of the SEM model, as seen in Fig. 2. Although the programme does not require family permission, some participants talked about the fact that parental authority held the most sway, which is a cultural and religious matter. It was crucial for participants to please their parents or family members, even after death, which is an idea deeply ingrained in their religious values. Pleasing parents was tied to being awarded heaven; therefore, their parents’ approval was essential, even if the respondents were married. In addition, some participants (especially Arabs) talked about family hierarchy, where parents hold the highest rank; hence, their permission has to be obtained, and their decisions are not always negotiable. Another important point was wanting to make sure that family members and parents were prepared for this to avoid having them experience intense emotions and react hysterically.

*‘Well, I will need to check with my parents. I mean you know how things go (Laughs). You know what? If I tell my mom about this, she will go mad’. (Participant 12, female, 41 years old)*

The identity of the recipient of organs was also important to participants: they were willing to donate only if the recipient was a family member, a friend, or a person sharing the same cultural or religious beliefs. However, this is not possible, given the nature of the programme.

*‘No, Unless a family member or a friend needs it’. (Participant 6, male, 22 years old)*

*‘I would probably do it if it went to Hindus only’. (Participant 16, female, 56 years old)*

#### Third theme: religious conviction

Religious conviction is another theme that emerged from all five SEM levels. Religion was an important factor in either encouraging or discouraging participants from registering for the deceased organ donation programme. One of the major issues reported was the lack of clear guidance on whether the process is acceptable from a religious perspective. Participants mentioned that they were unaware of the current religious outlook on deceased organ donation and that religious authorities should provide guidance about the current practice concerning end-of-life organ donation and reach a consensus, since deceased organ donation still seems to be an area of controversy in all religions. The respondents felt that the conflict arose from a clash between the values of charity and having to maintain body integrity by keeping it intact.

*‘What I know that Islam forbids organ donation, and do not allow another Muslim to receive organs. If there will be a clear FATWA from the head of FATWA, then I have NO issue in donating my organs’. (Participant 4, male, 39 years old)*

#### Fourth theme: knowledge and personal experience

Knowledge mainly stemmed from the individual and policy levels of the SEM. Most participants were not fully aware of all the details regarding the deceased organ donation programme, although they had heard about it. Many of the respondents made it clear that they had never thought about it because they felt it was not relevant to them, except for those who witnessed someone go through the experience. Their limited knowledge and awareness of the programme and the whole process seemed to have affected their decision to register for the programme. Although organ donation is very beneficial, most participants asked for further clarification, which indicates that knowledge about the subject was scarce. They also mentioned that the lack of campaigns to promote the programme might be an issue, especially since deciding to sign up for the programme is complicated by numerous factors, which have to be discussed in these promotion campaigns.

*‘Health authorities should talk about it, and there has to be more information from many bodies, at least the religious ones, how things will be done? How will families be informed? And what if they disagree?’ (Participant 14, male, 28 years old)*

Several participants reflected on their own experiences or the experiences of someone they knew with organ donation. In general, the respondents felt that this programme would be of great benefit because of their experiences or what they had observed while watching someone go through it. In other words, their experience or observation served as a source of knowledge on how useful this programme would be for many people.

*‘Yes, one of my best friends suffered for years from kidney failure and liver failure. And he went abroad to reach a kidney donor in China and to do the operation there. The liver donor was his son. He has taken half of his son’s liver and did the operation in Singapore. If this programme was available, he might not suffered this much’. (Participant 1, male, 53 years old)*

## Discussion

This study intended to explore the extent to which context plays a role in participants’ willingness to register for the deceased organ donation programme in Dubai, UAE. Although people’s perceptions about deceased organ donation have been studied before, they were mainly explored using a quantitative positivist approach [20, 44]. The positivist approach despite its advantages, it tend to reduce and simplify complex phenomenon such as the one explored in this study. Hence, does not enable engendering a holistic understanding of the phenomenon under investigation. Our findings brought to light that despite organ donation being a human and noble act, dehumanising the dignity of the body and the fear of pain, death, and the unknown are factors that prevent people from signing up for the programme. This result agrees with the literature, where participants regarded death as an ominous matter and avoided discussing it [45]. Along the same lines, the participants of this study were also concerned about the possibility of unethical acts related to withholding care or organ trafficking. These concerns are not new, as participants in a study by Kumar mentioned that they had not signed up for donor cards due to their fear that physicians might expedite their death to be eligible for organ donation [46]. Ralph et al. also mentioned that participants in the United States, United Kingdom, South Africa, and Spain questioned the standard of medical care provided to donors and had the same concerns regarding not trusting the organ donation process [10]. In fact, due to their mistrust in the allocation process, participants wanted to know the identity of the recipient [10], which is the exact same concern that emerged in our study. In the same vein, some other studies mentioned that African-American participants believed that ‘rich or famous’ individuals were more likely to be allocated organs than other patients [47] and that certain ethnic groups are racially discriminated against to mainly supply organs [48].

Obtaining the approval of family members and especially parents is of prime importance not only in this study, but in others as well [15, 49, 50]. As per our findings and data from the literature, there is a direct connection between the willingness to donate and family support [15, 49, 50]. In our study, participants felt that their parents’ approval was a must, irrespective of their age or even their marital status. These findings echoed Ralph et al.’s outcomes regarding parental approval [15]. This was mainly due to their religious beliefs, where parents’ blessings are tied to being awarded with heaven. In certain societies, such as the one in the UAE or the Middle East, parents hold the utmost power within the family; therefore, the rest of family members have to follow, even if this involves donating one’s organs as a good deed. In fact, some mothers strongly advocated that they should be the ones to make the decision about donating their child’s organs [51–53].

Religion and beliefs were considered major determinants in registering for the deceased organ donation programme. The absence of consensus from different religious authorities made participants hesitant and uncomfortable to sign up for the programme. The same effect has been seen in the literature, where some participants were uncertain about whether their religion supported donation, and therefore felt conflicted and uncertain about the subject [10, 15]. Various religions forbid violating the human body, whether living or dead [54]. However, they also place altruism and saving a life very highly [54–56]. Given this dilemma, many respondents were still hesitant: some saw organ donation as a gift to live [57] and that it is acceptable for people to donate their organs [45, 58], while others felt that retaining the body intact after death is a religious act, as in the case of our study [58].

Knowledge is another important factor, as not having a holistic understanding of organ donation due to the lack of knowledge seemed to impact the decision making. The public dissemination of information that addresses all segments of society could render the deceased organ donation programme more acceptable [49, 50]. The majority of participants thought that organ donation does not directly concern them, and similar findings were seen in the literature [15], which is a clear indication that raising awareness is crucial. Furthermore, technical knowledge about deceased organ donation was scarce amongst our participants, as found in previous studies [15], which further emphasises the importance of promotion campaigns and raising awareness. However, in the present work, the participants were keener on knowing about the religious outlook and how to educate their families and more. Hence, promotion and awareness campaigns have to be tailored to match the contexts of people.

Figure 2 clearly shows the various contextual factors that were identified by the participants to play a role in their decision to register for the deceased organ donation programme. These factors stemmed from various levels of the SEM. However, Fig. 2 also shows the interactive and dynamic character of the relationship between factors from various levels [21, 22]. This is a clear indication that these contextual factors are closely tied to each other; therefore, we should not treat these relationships as unidirectional. In addition, using the SEM while developing the topic guide helped to unravel data that enriched our understanding of laypersons’ actions in terms of their logic, knowledge, and beliefs grounded in the context of their daily lives [59]. To illustrate, the participants who talked about the need for parental approval were mainly concerned about their religious and cultural boundaries. Nevertheless, the subjects clearly stated that their lack of knowledge or misconceptions underpinned their fear. Hence, it is important to explore these factors in relation to each other and as a whole. To improve the organ donation culture in society, all SEM levels have to be considered during awareness campaigns and address people’s concerns, such as those unravelled by this study.

It is important to bear in mind that it is impossible to understand people’s perceptions towards organ donation without capturing their lay knowledge. The data that was collected during the interviews were merely a reflection on the programme in light of their unique contexts. This is important because it influences acceptability, since behaviour and perception are interconnected [60–68]. Perception does not only create an experience of the world, but it also allows us to act within contexts and environments [69]. Hence, participants’ lay knowledge regarding the deceased organ donation programme helped us understand what would affect people’s willingness in greater depth and breadth.

## Conclusion

The deceased organ donation programme has only recently been developed in the UAE. This qualitative study was conducted using a constructivist philosophical approach to gain insights into individuals’ personal beliefs, opinions, and perspectives. This helped provide a holistic understanding of the phenomenon of interest and identify future directions to render it a successful program. The responses of the participants shed light on the need for proper awareness campaigns about the programme. In addition, unless the religious view of deceased organ donation becomes clear and in favour of it, participation will be scarce. The social context should not be overlooked; for example, family ties in societies such as the UAE are essential and are found to play a pivotal role in deciding to donate an organ after death. This is not to say that these results are conclusive; however, this study provides a direction for future research on the important topic of deceased organ donation.

## Supplementary Information

**Additional file 1.** Topic guide used during the in semi-structured interviews.

## Data Availability

The datasets generated and/or analysed during the current study are not publicly available to comply with the Institutional Review Board of the Higher Colleges of Technology guidelines, but are available from the corresponding author on reasonable request.

## Abbreviations

UAE: United Arab Emirates
SEM: Social ecological model
