# Supplementary material for: How context affects people’s willingness to register for the deceased organ donation programme
Source: BMC Public Health. 2021 Apr 15;21:729. doi: 10.1186/s12889-021-10753-5 (PMC8048161; doi:10.1186/s12889-021-10753-5)
Supplement: Supplementary file 1 — Additional file 1. Topic guide used during the in semi-structured interviews. [file 12889_2021_10753_MOESM1_ESM.docx]

| **Interview Topic guide.** | | |
| --- | --- | --- |
| **Introduction of the interviewer** | | |
| My name is …………., am a senior pharmacy student/ assistant professor of pharmacy at the HCT. Thank you for agreeing to partake in our research. In this interview, our goal is to obtain more information about the lay public perception towards deceased organ donation in UAE, and how context affects their willingness to sign up for the programme.  The interview should take 45 minutes maximum, and it will be audio recorded, the record will start only after obtaining your demographic information, therefore your identity will remain anonymous.  Remember, there are no right or wrong answers, and your replies will be anonymized only.  Are you ready to respond to some questions currently? | | |
| **General demographics information** | | |
| - Name - Age - Gender - Professional - Nationality - Religion | | |
| Code number: ………… (To substitute names for anonymity purposes) | | |
| **starter questions** | | |
| **Main questions** | **Additional questions** | **Clarifying questions/ Probes** |
| - Have you ever considered deceased organ donation? - To what extent do you know about deceased organ donation and how have you come to know about it? - What do you know about the National Program for Organ Donation in the UAE? (If they don’t know, explain it.) | - Why? - When? - What made you think about it? | - Can you expand a little on this? - Can you tell me anything else? - Can you give me some examples? |
| **Perceptions and beliefs** | | |
| **Main questions** | **Additional questions** | **Clarifying questions/ Probes** |
| - Why would/ wouldn’t you register for deceased organ donation? - What is your biggest fear regarding organ donation? - If I had the deceased organ donation registration papers, would you register for it? Why, why not? - Do you think this programme will gain popularity? Why/ why not? | - Why? Why not? - What is affecting your decision? - What are you thinking about? | - Can you expand a little on this? - Can you tell me anything else? - Can you give me some examples? |
| **CONCLUSION:** Well, this concludes our interview. It has been a pleasure meeting you today. I appreciate you taking the time out of your schedule to come and show your generous support. I would like to remind you that you have 2 weeks from today to withdraw your data, please contact me at any time if you feel you need to discuss this further (business card provided). Thank you again for your time, input and presence. | | |
